# Lifetime body mass index and risk of oral cavity and oropharyngeal cancer by smoking and drinking habits

**DOI:** 10.1038/sj.bjc.6601347

**Published:** 2003-10-28

**Authors:** A Nieto, M J Sánchez, C Martínez, X Castellsagué, M J Quintana, X Bosch, M Conde, N Muñoz, R Herrero, S Franceschi

**Affiliations:** 1 Departamento de Ciencias Sociosanitarias, Facultad de Medicina, Universidad de Sevilla, Avda. Sánchez Pizjuan S/n, 41009 Sevilla, Spain; 2 Escuela Andaluza de Salud Pública, Granada, Spain; 3 Institut Català d'Oncologia, Barcelona, Spain; 4 International Agency for Research on Cancer, Lyon, France; 5 Proyecto Epidemiológico Guanacaste, Costa Rican Foundation for Health Sciences, San José, Costa Rica

**Keywords:** alcohol, body mass index, cancer of oral cavity and oropharynx, leanness, smoking

## Abstract

The influence of body mass index (BMI) on oral cancer risk was evaluated in 375 incident cases and 375 age–gender-matched hospital-based controls. Low BMIs at diagnosis and 2 years before diagnosis were associated with significantly elevated odds ratios (OR for BMI ⩽22 *vs* >26 kg m^−2^; 3.64; 95% confidence interval, CI: 2.27–5.82 and 3.31; 95% CI: 2.04–5.39, respectively). The association with low BMI, however, tended to be weaker and nonsignificant among never smokers and never drinkers.

Mortality and incidence of cancers of the oral cavity and oropharynx (oral cancer) have been increasing in many areas of the world in recent decades (Parkin *et al*, 1999; Pisani *et al*, 1999), most notably in young adults (Levi *et al*, 1999; Mackenzie *et al*, 2000; Llewellyn *et al* (2001)).

Risk factors for oral cancer include tobacco smoking and alcohol consumption (Blot *et al*, 1988) and low intake of fruit and vegetables (Bosetti *et al*, 2000; Tavani *et al*, 2001). Recently, the role of other factors, such as poor oral hygiene and dentition (Talamini *et al*, 2000), genetic susceptibility (Warnakulasuriya *et al*, 1998), sexual habits (Garrote *et al*, 2001) and infection by human papillomavirus (Mork *et al*, 2001), has been investigated.

The aim of this report is to assess whether body mass index (BMI) estimated at various points in life is related to oral cancer after taking into account the influence of established aetiological factors.

## MATERIAL AND METHODS

The present case–control study is part of an international study on oral cancer and human papillomavirus coordinated by the International Agency for Research on Cancer (IARC) (Herrero *et al*, in press) and carried out simultaneously in Spain, Italy, Ireland, Poland, Cuba, Canada, India, Sudan and Australia. The project was approved by the Ethical Committee of IARC and local research and ethical committees. Informed consent was obtained from each participant in the study.

Eligible cases were incident, histologically confirmed and consecutively diagnosed (between 1996 and 1999) invasive cancers of the oral cavity and oropharynx, codes C 01–C 10 of the second edition of the International Classification of Diseases for Oncology (Percy *et al*, 1990) from four hospitals: two in Barcelona, one in Granada and one in Seville. The overall participation among eligible cases was 76.5% (375 out of 490): 70.6% in Barcelona, 78.2% in Granada and 90.5% in Seville.

Controls were in-patients or outpatients in the same hospitals with conditions unrelated to smoking, alcohol or long-term modification of diet. They were frequency-matched with cases by age (in 5-year periods), gender and hospital. One control was selected for each case in the 3-month time interval after the recruitment of the case. The overall participation among controls was 91% (375 out of 412): 91.1% in Barcelona, 91.2% in Granada and 90.5% in Seville. Exfoliated cells from oral cavity, blood samples and tissue biopsies were also collected from cases and controls.

Identical questionnaires and coding manuals were used in each centre, and all interviewers received the same training and were routinely supervised. Body mass indexes were calculated from self-reported height and weight at diagnosis, 2 years before diagnosis, and in young adulthood, as weight in kilograms divided by the square of height in metres. A difference between the questionnaires used in the three centres was that participants in Barcelona were asked to report their weight at the age of 30 years, while in Granada and Sevilla they reported their weight at the age of 20 years; both are referred to herein as weight in young adulthood. Height, weight and BMI distributions were categorised into approximate tertiles (based on the whole distribution of participants) using the highest tertile as the reference category.

Smoking was defined as having smoked at least one cigarette (or the equivalent) daily for at least 1 year. Subjects were also asked about duration of the habit and amount of tobacco smoked. Drinking was defined as having drunk alcoholic beverages at least once a month, and details on amount and duration were obtained.

Odds ratios (ORs) and corresponding 95% confidence intervals (CIs) were calculated for the overall number of participants using unconditional multiple logistic regression models (Breslow and Day, 1980) that included as covariates the three design variables: age, gender and centre, and also years of schooling, as an indicator of social and economic status. Odds ratios were further adjusted for: average number of cigarettes smoked per day, average millilitres of ethanol consumed per day and weekly consumption of fruit and vegetables. All these variables were categorized in approximate quartiles of the whole distribution of participants.

Dose–response relationship with risk was evaluated by treating categorical ordinal variables as continuous in the logistic regression models. All *P*-values were derived from two-sided statistical tests.

## RESULTS

Table 1

**Table 1:** Sociodemographics characteristics, smoking and alcohol drinking status, fruit and vegetables intake in cases and controls, Spain, 1996–1999

	**Males**		**Females**	
	**Cases^a^**	**Controls^a^**	**Cases^a^**	**Controls^a^**
	***N* (%)**	***N* (%)**	***N* (%)**	***N* (%)**
*Age groups (years)*				
20–51	77 (25.3)	76 (25.0)	21 (29.6)	18 (25.4)
52–59	76 (25.0)	78 (25.7)	9 (12.7)	10 (14.1)
60–69	91 (29.9)	90 (29.6)	12 (16.9)	13 (18.3)
⩾70	60 (19.8)	60 (19.7)	29 (40.8)	30 (42.2)
*Years of schooling*				
0	35 (11.5)	37 (12.2)	8 (11.3)	9 (12.7)
1–5	90 (29.6)	92 (30.3)	20 (28.2)	16 (22.5)
6–8	89 (29.3)	88 (28.9)	20 (28.2)	28 (39.4)
⩾9	89 (29.3)	87 (28.6)	22 (31.0)	18 (25.4)
*Smoking status*				
Never smokers	5 (1.6)	52 (17.1)	50 (70.4)	61 (85.9)
Exsmokers	76 (25.0)	130 (42.8)	6 (8.5)	3 (4.2)
Current smokers	223 (73.4)	122 (40.1)	15 (21.1)	7 (9.9)
*Alcohol drinking status*				
Nondrinkers	4 (1.3)	27 (8.9)	31 (43.7)	48 (67.6)
Exdrinkers	74 (24.3)	95 (31.2)	15 (21.1)	8 (11.3)
Current drinkers	226 (74.4)	182 (59.9)	25 (35.2)	15 (21.1)
*Vegetables intake (portions)*				
⩽3	124 (40.8)	93 (30.6)	19 (26.8)	27 (38.0)
4–7	129 (42.4)	127 (41.8)	35 (49.3)	23 (32.4)
⩾8	45 (14.8)	82 (27.0)	16 (22.5)	21 (29.6)
*Fruits intake (pieces)*				
⩽5	134 (44.1)	74 (24.3)	15 (21.1)	19 (26.8)
6–9	90 (29.6)	112 (36.8)	28 (39.4)	24 (33.8)
⩾10	75 (24.7)	116 (38.2)	27 (38.0)	28 (39.4)
				
*Number of subjects*	304 (100)	304 (100)	71 (100)	71 (100)

a Some strata do not add up to the total because of a few missing values.

shows the distribution of study participants by gender and age, years of schooling, smoking and drinking habits, and intake of fruit and vegetables. A marked excess of current smokers and alcohol drinkers was found among cases compared to controls of each gender. After adjusting for smoking and drinking habits, there were no differences between cases and controls in years of schooling (data not shown).

Table 2

**Table 2:** Height, weight and body mass index: odds ratios (OR) and confidence intervals (95% CI) for oral cavity and oropharyngeal cancer, Spain 1996–1999

	**Cases^a^**	**Controls^a^**	**OR^b^**	**95% CI**
*Height (cm)*				
⩾170	101	124	1.0^c^	
169–163	113	116	1.19	0.78–1.82
⩽162	123	97	1.39	0.88–2.19
* P*-value for trend			0.103	
**At diagnosis**				
*Weight (kg)*				
⩾76	75	147	1.0^c^	
75–66	87	123	1.20	0.78–1.86
⩽65	179	75	3.57	2.32–5.51
* P*-value for trend			**<0.001**	
* Body mass index (kg m^−2^)*				
⩾26	113	204	1.0^c^	
25–23	104	88	1.84	1.22–2.76
⩽22	112	40	3.64	2.27–5.82
* P*-value for trend			**<0.001**	
				
** 2 years before**				
* Weight (kg)*				
⩾76	80	149	1.0^c^	
75–66	108	126	1.32	0.87–2.00
⩽65	159	79	2.96	1.93–4.53
*P*-value for trend			**<0.001**	
*Body mass index (kg m^−2^)*				
⩾26	129	207	1.0^c^	
25–23	106	88	1.65	1.11–2.47
⩽22	96	36	3.31	2.04–5.39
*P*-value for trend			**<0.001**	
				
**In young adulthood**				
* Weight (kg)*				
⩾69	98	135	1.0^c^	
68–61	90	91	1.27	0.82–1.97
⩽60	155	125	1.49	0.99–2.26
*P*-value for trend			0.065	
*Body mass index (kg m^−2^)*				
⩾25	100	118	1.0^c^	
24–23	85	94	0.98	0.63–1.53
⩽22	140	113	1.32	0.87–2.00
*P*-value for trend			0.211	

a Some strata do not add up to the total because of missing values.

b Estimates from unconditional regression equations including terms for age, gender, centre, education, smoking and drinking habits, fruits and vegetables intakes.

c Reference category.

shows a significant association between oral cancer risk and low BMI (⩽22 *vs* ⩾26 kg m^−2^) at diagnosis (OR: 3.64; 95% CI: 2.27–5.82), and 2 years before diagnosis (OR: 3.31; 95% CI: 2.04–5.39; *P*<0.001) after adjusting for smoking, drinking and the intake of fruit and vegetables. Conversely, low BMI in young adulthood (⩽22 *vs* ⩾25 kg m^−2^) was not significantly related to oral cancer risk (OR: 1.32; 95% CI: 0.87–2.00).

The same variables are re-evaluated in Tables 3

**Table 3:** Height, weight and body mass index in never, former and current smokers: Odds ratios (OR) and confidence intervals (95% CI) for oral cavity and oropharyngeal cancer, Spain 1996–1999

	**Never smokers**			**Former smokers**			**Current smokers**		
	**Ca/Co^a^**	**OR**^b^	**95% CI**	**Ca/Co^a^**	**OR^c^**	**95% CI**	**Ca/Co^a^**	**OR^c^**	**95% CI**
*Height (cm)*									
⩾170	4/21	1.0^d^		22/52	1.0^d^		75/51	1.0^d^	
169–163	7/32	0.14	0.01–1.68	29/41	1.79	0.78–4.10	77/43	1.43	0.76–2.70
⩽162	29/41	0.24	0.02–3.12	20/34	1.73	0.69–4.33	74/22	1.88	0.91–3.86
*P*-value for trend		0.932			0.192			0.062	
									
**At diagnosis**									
*Weight (kg)*									
⩾76	12/40	1.0^d^		25/54	1.0^d^		38/53	1.0^d^	
66–75	11/31	1.44	0.46–4.49	20/50	0.70	0.28–1.74	56/42	1.67	0.85–3.27
⩽65	24/28	2.28	0.79–6.59	28/21	3.60	1.45–8.94	127/26	4.57	2.29–9.09
*P*-value for trend		0.096			**0.009**			**<0.001**	
									
*Body mass index (kg m^−2^)*									
⩾26	21/59	1.0^d^		31/81	1.0^d^		61/64	1.0^d^	
23–25	11/25	1.12	0.38–3.28	25/28	2.34	1.02–5.35	68/35	1.59	0.84–3.02
⩽22	7/9	3.45	0.85–14.1	15/15	2.89	1.07–7.80	90/16	4.12	1.99–8.51
* P*-value for trend		0.191			**0.011**			**<0.001**	
									
**2 years before**									
* Weight (kg)*									
⩾76	11/40	1.0^d^		24/59	1.0^d^		45/50	1.0^d^	
75–66	13/31	1.92	0.64–5.80	24/49	1.12	0.48–2.63	71/46	1.40	0.73–2.69
⩽65	25/32	2.71	0.94–7.85	22/21	2.54	1.02–6.35	112/26	3.51	1.77–6.96
*P*-value for trend		0.068			**0.047**			**<0.001**	
									
*Body mass index (kg m^−2^)*									
⩾26	20/59	1.0^d^		34/84	1.0^d^		75/64	1.0^d^	
25–23	14/24	2.23	0.76–6.40	25/30	1.69	0.78–3.69	67/34	1.31	0.69–2.48
⩽22	5/9	2.38	0.53–10.7	10/11	1.72	0.56–5.30	81/16	4.10	1.97–8.55
*P*-value for trend		0.134			0.163			**<0.001**	
									
**In young adulthood**									
*Weight (kg)*									
⩾69	12/32	1.0^d^		22/54	1.0^d^		64/49	1.0^d^	
68–61	8/17	1.11	0.27–4.56	23/33	2.15	0.88–5.27	59/41	1.15	0.59–2.24
⩽60	30/55	0.78	0.28–2.18	25/39	1.68	0.69–4.12	100/31	2.57	1.31–5.06
*P*-value for trend		0.569			0.229			**0.007**	
									
*Body mass index (kg m^−2^)*									
⩾25	14/34	1.0^d^		20/43	1.0^d^		66/41	1.0^d^	
24–23	7/18	1.26	0.33–4.76	20/39	1.20	0.49–2.94	58/37	1.00	0.51–1.98
⩽22	17/40	0.99	0.37–2.69	29/40	1.34	0.56–3.21	94/33	2.13	1.07–4.24
*P*-value for trend		0.922			0.514			**0.041**	

a Some strata do not add up to the total because of a few missing values.

b Estimates from unconditional regression equations including terms for age, gender, centre, education, drinking habits, fruit and vegetables intake in never smokers.

c Estimates from unconditional regression equations including terms for age, gender, centre, education, smoking and drinking habits, fruit and vegetables intakes in former and current smokers.

d Reference category.

and 4

**Table 4:** Height, weight and body mass index in never and ever alcohol drinking: odds ratios (OR) and confidence intervals (95% CI) for oral cavity and oropharyngeal cancer, Spain 1996–1999

	**Nondrinkers**			**Ever-drinkers**		
	**Ca/Co^a^**	**OR^b^**	**95% CI**	**Ca/Co^a^**	**OR^c^**	**95% CI**
*Height (cm)*						
⩾170	2/14	1.0^d^		99/110	1.0^d^	
169–163	5/16	1.70	0.16–18.25	108/100	1.22	0.78–1.89
⩽162	16/28	2.50	0.18–34.54	107/69	1.43	0.88–2.33
*P*-value for trend		0.449			0.100	
						
***At diagnosis***						
*Weight (kg)*						
⩾76	6/25	1.0^d^		69/122	1.0^d^	
66–75	5/17	2.70	0.49–14.84	82/106	1.18	0.75–1.87
⩽65	15/19	3.74	0.92–15.19	164/56	3.54	2.21–5.66
*P*-value for trend		**0.047**			**<0.001**	
*Body mass index (kg m^−2^)*						
⩾26	10/33	1.0^d^		103/171	1.0^d^	
23–25	6/16	2.11	0.51–8.77	98/72	1.82	1.17–2.81
⩽22	6/8	2.06	0.42–10.22	106/32	3.64	2.18–6.05
* P*-value for trend		**0.495**			**<0.001**	
						
**2 years before**						
*Weight (kg)*						
⩾76	6/26	1.0^d^		74/123	1.0^d^	
75–66	8/19	2.97	0.61–14.49	100/107	1.22	0.78–1.92
⩽65	14//20	3.32	0.81–13.64	145/59	2.90	1.82–4.61
* P*-value for trend		**0.098**			**<0.001**	
						
*Body mass index (kg m^−2^)*						
⩾26	13/37	1.0^d^		116/170	1.0^d^	
25–23	6/12	4.06	0.89–18.49	100/76	1.59	1.04–2.43
⩽22	4/7	1.26	0.23–6.87	92/29	3.39	2.00–5.73
*P*-value for trend		**0.419**			**<0.001**	
						
**In young adulthood**						
* Weight (kg)*						
⩾69	5/24	1.0^d^		93/111	1.0^d^	
68–61	6/11	4.53	0.69–29.69	84/80	1.15	0.72–1.83
⩽60	19/30	3.89	0.80–18.77	136/95	1.45	0.93–2.27
*P*-value for trend		**0.238**			0.102	
						
*Body mass index (kg m^−2^)*						
⩾25	8/22	1.0^d^		92/96	1.0^d^	
24–23	5/15	1.98	0.38–10.39	80/79	0.90	0.56–1.46
⩽22	9/18	1.37	0.36–5.27	131/95	1.33	0.85–2.09
*P*-value for trend		**0.920**			0.237	

a Some strata do not add up to the total because of a few missing values.

b Estimates from unconditional regression equations including terms for age, gender, centre, education, smoking habits, fruits and vegetables intake in nondrinkers.

c Estimates from unconditional regression equations including terms for age, gender, centre, education, smoking and drinking habits, fruits and vegetables intakes in ever-drinkers.

d Reference category.

in separate smoking and drinking strata. A significant association was found between risk and low BMI at diagnosis (OR: 4.12; 95% CI: 1.99–8.51; *P*<0.001) and 2 years before diagnosis (OR: 4.10; 95% CI: 1.97–8.55; *P*<0.001) among current smokers (Table 3). Low BMI in young adulthood was also associated with an increased risk of oral cancer in current smokers (OR: 2.13; 95% CI: 1.07–4.24; *P*=0.041). A similar relationship was observed in former smokers only for BMI at diagnosis (OR: 2.89; 95% CI: 1.07–7.80; *P*=0.011), but was not present in never smokers.

Low BMI at diagnosis (OR: 3.64; 95% CI: 2.18–6.05; *P*<0.001), and 2 years before diagnosis (OR: 3.39; 95% CI: 2.00–5.73; *P*<0.001) were related to an increased risk of oral cancer among ever-drinkers, but not among never drinkers (Table 4).

## DISCUSSION

Our results are consistent with some previous studies in showing an inverse association between leanness and increased oral cancer risk (Kabat *et al*, 1994; Franceschi *et al*, 2001). Subjects were asked about their weight 2 years before diagnosis and in young adulthood in an attempt to evaluate weight before the diagnosis of cancer. Obviously the choice of any specific life period is somewhat arbitrary since the time at which oral cancer started is unknown (Hawkins *et al*, 1999).

As with most case–control studies, our study may be affected by bias and confounding. In particular, the selection of hospital controls is open to criticism. However, we chose as controls individuals who, in the event of a cancer diagnosis, would have been admitted to the same hospitals where the cases had been identified (Rothman, 2002). Another possible weakness is reliance on self-reported weight and height. The absence of a relationship between BMI in young adulthood and subsequent risk of oral cancer might have been influenced by this long time of recall.

We observed a gradual decrease in the magnitude of oral cancer risk from current smokers to former smokers and to nonsmokers. In fact, the influence of leanness on risk lacked significance in nonsmokers, and it was statistically significant in former smokers only at diagnosis, but not 2 years before diagnosis. In contrast, among current smokers, leanness was strongly associated with an increased risk at any one point in time, suggesting that smokers, in addition to being exposed to a high level of carcinogens, may suffer from weight loss as an expression of nutritional deficiency (Franceschi *et al*, 2001). Low BMI might therefore be a result of smoking. The Minnesota Lipid Research Clinic (LRC) Prevalence Study showed, however, that smokers of 15–29 cig/day generally consumed at least as many or more calories as those who had never smoked yet had lower weight (Jacobs and Gottenborg, 1981).

Similarly, it has been shown that ethanol may contribute to this effect by altering absorption and metabolism of many different nutrients (Clinton *et al*, 2000). High ethanol intake causes primary malnutrition by replacing nutrients in the diet and secondary malnutrition via malabsorption and cellular injury (Lieber, 1993). Additionally, excessive consumption of alcoholic beverages is often associated with poor dietary habits (Darmon *et al*, 2001). In our study, leanness at diagnosis and 2 years before diagnosis was significantly associated with an increased risk among drinkers, but not in nondrinkers. Overall, however, a strong effect of low BMI on oral cancer risk was confirmed after careful adjustment for smoking, alcohol and dietary habits.
